# Gene and Protein Expression following Exposure to Radiofrequency Fields from Mobile Phones

**DOI:** 10.1289/ehp.11279

**Published:** 2008-05-13

**Authors:** Jacques Vanderstraeten, Luc Verschaeve

**Affiliations:** 1 Research Unit on Work Health and Environmental Toxicology, School of Public Health, Université Libre de Bruxelles, Brussels, Belgium; 2 Department of Toxicology, Scientific Institute of Public Health, Brussels, Belgium

**Keywords:** gene expression, genomics, microwaves, mobile phones, protein expression, proteomics, radiofrequency fields

## Abstract

**Background:**

Since 1999, several articles have been published on genome-wide and/or proteome-wide response after exposure to radiofrequency (RF) fields whose signal and intensities were similar to or typical of those of currently used mobile telephones. These studies were performed using powerful high-throughput screening techniques (HTSTs) of transcriptomics and/or proteomics, which allow for the simultaneous screening of the expression of thousands of genes or proteins.

**Objectives:**

We reviewed these HTST-based studies and compared the results with currently accepted concepts about the effects of RF fields on gene expression. In this article we also discuss these last in light of the recent concept of microwave-assisted chemistry.

**Discussion:**

To date, the results of HTST-based studies of transcriptomics and/or proteomics after exposure to RF fields relevant to human exposure are still inconclusive, as most of the positive reports are flawed by methodologic imperfections or shortcomings. In addition, when positive findings were reported, no precise response pattern could be identified in a reproducible way. In particular, results from HTST studies tend to exclude the role of a cell stressor for exposure to RF fields at nonthermal intensities. However, on the basis of lessons from microwave-assisted chemistry, we can assume that RF fields might affect heat-sensitive gene or protein expression to an extent larger than would be predicted from temperature change only. But in all likelihood, this would concern intensities higher than those relevant to usual human exposure.

**Conclusions:**

The precise role of transcriptomics and proteomics in the screening of bioeffects from exposure to RF fields from mobile phones is still uncertain in view of the lack of positively identified phenotypic change and the lack of theoretical, as well as experimental, arguments for specific gene and/or protein response patterns after this kind of exposure.

The widespread use of cellular telephones has aroused public concern with respect to potential health risks associated with the radio-frequency (RF) fields emitted by these devices and their base station antennas. Although it is still too soon to assess with certainty or to definitively rule out possible long-term effects, experimental as well as epidemiologic studies to date have not provided any solid indication in favor of the presence or absence of a mobile communication-related health problem (Cardis et al. 2007; Valberg et al. 2007). The INTERPHONE study, a multinational case–control study, has raised concern about possible increased prevalence of acoustic neuroma among regular users of mobile phones for > 10 years (Cardis et al. 2007). However, questions have been raised about possible errors in the estimation of this risk due to recall and participation biases (Cardis et al. 2007). Several *in vivo* studies addressed possible tumorigenicity in animals (mostly rats and mice) exposed for extended periods (up to lifetime exposures of 2 years) and at a variety of modulations and frequencies (435–9,400 MHz). Results were inconclusive. Only three tests (20%) in the 15 studies addressing continuous waves (CWs) and four tests (6%) in 63 studies addressing modulated fields were positive. Overall, consistency and reproducibility were lacking (Valberg et al. 2007). To date, a number of *in vitro* investigations of RF-induced genetic effects in human and other cell types have been conducted, most of which have indicated no evidence of *in vitro* RF-induced genetic damage at nonthermal exposure regimes or marked synergistic or additive effect with another environmental agent (mutagen/carcinogen) (Meltz 2003; Verschaeve 2005; Vijayalaxmi and Obe 2004). In addition, some investigators have suggested that RF fields may act as a cancer promoter; however, the evidence for a cocarcinogenic effect or an effect on tumor promotion or progression is at most suggestive, not substantive (Bartsch et al. 2002; Mason et al. 2001).

The majority of the studies were devoted to genetic end points that correspond to genotoxic or mutational changes unlikely to occur at exposure intensities relevant to human exposure (Valberg et al. 2007). The investigation of the possibility of more subtle or functional effects on the transcription of genes, for example, requires the use of more sensitive methods, including gene and protein expression studies.

## Gene expression studies

If RF radiation at intensities relevant to human exposure produces any biological effect, this result must notably imply changes in cell behavior and changes in gene and protein expression in particular. Genes known to be stress-responsive (heat shock and immediate early genes) have been investigated most frequently since the first publications by Daniells et al. (1998) and de Pomerai et al. (2000) regarding heat shock gene expression in *Caenorhabditis elegans* after exposure to microwaves (MWs; RF fields > 300 MHz) at intensities too low to elicit any measurable temperature change. Cotgreave (2005) recently reviewed the studies addressing the expression of specific genes after RF exposures relevant to human mobile phone use. The author considered the results to be inconclusive and found that most positive findings were flawed by inconsistencies and lack in reproducibility. Since then, a number of studies have failed to show consistent effect on expression of either heat shock or immediate early genes from RF exposure [brief review in Chauhan et al. (2007) and Qutob et al. (2006)].

For almost a decade, high-throughput screening techniques (HTSTs) have been developed, allowing genome- and proteome-wide investigations of gene expression (functional genomics or transcriptomics) and protein expression (proteomics). These HTSTs are currently widely applied, notably in the field of environmental health sciences, with the objective of identifying possible markers of toxic exposure by discerning reproducible response patterns (Freeman 2005). Since the first work by Harvey and French (1999), several researchers have used HTSTs for investigation of gene and/or protein expression after RF exposure from mobile phones.

## Genome- and proteome-wide studies

Most transcriptomics applied to RF exposure use a microarray technique based on mRNA extraction and subsequent hybridization to cDNA or oligonucleotide probes representing numerous well-characterized genes. Proteomics has been investigated by HTST with the use of two-dimensional gel electrophoresis. These HTSTs allow for the simultaneous screening of the expression of thousands of genes or proteins, but because of their high sensitivity, they are prone to false-positive results (Leszczynski and Meltz 2006). It is important to take into account the statistical issues that are crucial for the interpretation of data (Mayo et al. 2006) and the confirmation experiments with quantitative tests that are required to evaluate positive findings. This is especially true, for example, when determining the significance of small changes (< 1.5- to 2.0-fold) in probed mRNA and protein levels. In addition, because of the variability of results between experiments related to experimental setup and methodology, another objective is the identification of biomarkers that are notably independent of technology platform (Corvi et al. 2006).

Table 1 provides a summary of the HTST studies of gene and/or protein expression after RF exposure that were published between 1999 and November 2007 in English. The signals used were either CWs or pulse-modulated waves (PMWs) of commercially used signals. Fifteen of the 17 reviewed studies used exposure signals and intensities similar to or typical of human exposure to mobile phone radiation; these studies used the signals of the code-division or frequency-division multiple access (CDMA or FDMA, respectively) system, of the PMWs of the Canadian mobile phone system, or of the time-division multiple access of the global system for mobile communications (GSM). The specific absorption rate (SAR) was between 0.1 and 10 W/kg, and the temperature conditions were generally well controlled. Two other investigations addressed gene expression after exposure to different signals and intensities: Harvey and French (1999) reported changes in 3 of 558 screened genes under intermittent exposure to CWs at an SAR of 7.3 W/kg but in only twice-repeated experiments and without quantitative confirmation experiments such as reverse transcriptase polymerase chain reaction (RT-PCR). In contrast, Port et al. (2003) observed no change under exposure to a 400-MHz pulsed E-field of 50 kV/m, but they used only a 6-min exposure and they did not mention pulse duration or SAR value.

From the 15 studies relevant to human exposure, 13 were related to gene expression. Of these, 4 studies that reported positive results were based only on single experiments. From these 4 studies, two reported positive findings that were not validated by confirmation experiments: Pacini et al. (2002) reported changes in the expression of 14 genes but with a rather imprecise exposure methodology, and Lee et al. (2005) reported changes in hundreds of genes after either 2-hr or 6-hr exposure (2.45 GHz PMW, 10 W/kg) but with no sham-controlled sample for the 6-hr exposure. Interestingly, and unlike all of the other researchers, Lee et al. (2005) used the serial analysis of gene expression (SAGE) technique for the screening of gene expression, and they fixed the cutoff value of fold change at a rather high value (4.0). With the use of oligonucleotide chip-based microarray, the two remaining studies reported positive findings that were further confirmed by RT-PCR: two responding genes among 8,400 screened (Gurisik et al. 2006) and 34 responding genes but with a cutoff value of only 1.15-fold change (Zhao et al. 2007).

Using membrane-based cDNA micro-array, the Bio-NIR Research Group of STUK (Radiation and Nuclear Safety Authority) in Finland published a total of three studies of gene expression in human endothelial (EA.hy926) cells with positive findings not yet confirmed by further quantitative experiment: *a*) down-regulation of various genes from the Fas/tumor necrosis factor-α (Fas/TNF-α) aptoptotic pathway but without precision of the method followed for the data analysis and interpretation (Leszczynski et al. 2004); *b*) 1 (EA.hy926 cells) and 13 (EA.hy926v1 cells) responding genes with a cutoff value of 2.0-fold change (Nylund and Leszczynski 2006); and *c*) 32 differentially expressed genes after 900-MHz RF exposure (Remondini et al. 2006). Remondini et al. (2006) reported the results of a multicentric study that was part of the REFLEX project (Quality of Life and Management of Living Resources 2004) of the European Union’s Fifth Framework Programme, and they also reported the results of five other research teams who studied different types of human cells after exposure to GSM 900 or 1,800 radiation with use of membrane-based cDNA microarray and with application of strict methods for false discovery rate (FDR) control. Although no change could be observed in three types of cells, the three other types (among which were EA.hy926 cells) showed consistent gene responses. However, the replicate experiments were technical ones (pooled RNAs), and no quantitative experiments were reported to validate the positive results (1.2-fold change considered). In addition, the interpretation of the positive results was rendered difficult by the variability in the gene response, notably in HL-60 cells under intermittent versus continuous exposure to 1,800-MHz fields or in EA.hy926 cells under exposure to 900-MHz versus 1,800-MHz fields. Two other studies also reported positive findings, which were not confirmed by quantitative experiments: 12 responding genes among 8,800 screened by oligonucleotide chip-based microarray (Belyaev et al. 2006) and 68 responding genes among 30,000 screened by glass cDNA microarray, for a SAR value of 10 W/kg (Huang et al. 2006).

The four remaining studies reported negative findings with use of a oligonucleotide chip-based microarray. Three of these studies used stringent methods for the preprocessing and analysis of data and for FDR control (they used positive and negative controls) (Chauhan et al. 2007; Qutob et al. 2006; Whitehead et al. 2006). In constrast, after having conducted an RT-PCR test, Zeng et al. (2006) could not confirm the positive results they found in DNA microarray screening.

Five articles addressed proteomic outcomes. The Bio-NIR Research Group of STUK reported positive findings in EA.hy926 cells in four different articles (Leszczynski et al. 2002, 2004; Nylund and Leszczynski 2004, 2006). In the first study, Leszczynski et al. (2002) addressed protein phosphorylation status with observation of hundreds of differentially phosphorylated proteins, but no repeat experiments were conducted; however, confirmation by two independent tests was obtained with respect to the increased phosphorylation level of heat shock protein (HSP) 27. The three other studies (Leszczynski et al. 2004; Nylund and Leszczynski 2004, 2006) addressed protein expression, all reporting positive findings in as many as 10 repeated independent experiments. An isoform of vimentin, a cytoskeleton protein, and other proteins were validated by further confirmation experiments. The remaining study on proteomics from Zeng et al. (2006) reported findings whose inconsistencies led the authors to believe that the observed changes might have occurred by chance.

In summary, the currently available results from studies of transcriptomics show a variability that must be due at least in part to the variability of the platform used and the methodology for data analysis and interpretation. This variability justifies the recommendations of Corvi et al. (2006) for standardization of methods. It cannot be ruled out that the variability of the results also reflects other specifics, such as the biosystem used or the characteristics of exposure, or the time point to assess bioeffect. However, most of the reported positive findings are flawed by methodologic imperfections or shortcomings and consequently need to be reproduced and validated by further confirmation experiments. Moreover, no specific pattern could be observed in a reproducible way in gene responses, even with the use of the same biosystems and/or experimental setup. In particular, results from HTST studies tend to exclude a role of cell stressor for exposure to RF fields at nonthermal intensities; with exposure to a cellular phone, an SAR of 1.6 W/kg causes a temperature elevation in head tissues of ≤ 0.2–0.3°C (Van Leeuwen et al. 1999). Furthermore, no mechanism has been identified for the activation of the heat shock gene expression in mammals by RF exposure at nonthermal intensity, which depends on heat-activated conformational change of the heat shock factor (HSF) protein (Morimoto 1998). Laszlo et al. (2005), for example, did not observe HSF activation in mammalian cells after exposure to FDMA- or CDMA-modulated RF fields, even at 5 W/kg.

From the few studies using HTSTs for proteomics, consistent protein responses were reported by Leszczynski et al. (2002, 2004) and Nylund and Leszczynski (2004, 2006). However, these observations should still be independently reproduced.

## Discussion

Overall, the results from studies of gene and protein expression after exposure to RF fields from mobile phones are inconclusive to date. Two questions should be asked concerning the use of HTSTs for the detection of possible health effects from exposure to RF fields from mobile phones. First, would a response, if confirmed, give evidence in favor of a risk of toxicity? This might not be the case, in principle, as long as no phenotypic change could be confirmed either *in vitro* or *in vivo* after RF exposure at intensities relevant to human exposure. In this respect, some authors simultaneously studied genomics and/or proteomics together with other related biological end points. Leszczynski et al. (2004), for example, observed an increase in HSP27 phosphorylation—which can be considered an antiapoptotic event (Morimoto 1998)—and a concomitant increase in stability of stress fibers of cells and down-regulation of genes of the Fas/TNF-α apoptotic pathway. Other authors did not observe simultaneous change in gene expression and in related downstream events: Natarajan et al. (2006) reported increased DNA-binding activity of NF-kappaB in human monocytes but no transactivation of kappaB-dependent gene expression after exposure to pulsed ultra-wide-band electromagnetic fields. Nikolova et al. (2005) observed changes in gene expression but without detectable change in cell physiology. Nylund and Leszczynski (2006) observed simultaneous changes in gene expression and in protein expression, but the latter were not related to the former.

Second, are HTSTs a relevant method here? In other words, could a specific gene or protein response pattern ever be established after exposure to RF fields from mobile phones? From the strict point of view of biophysics, an effect of RF fields emitted by current cellular phones on gene and/or protein expression is unlikely to occur as long as no mechanism can be identified for the interaction between these fields and living tissues, with the exception of the known conversion of electromagnetic energy into thermal energy. Yet, whatever the exposure intensity, the possibility exists that RF fields influence these biochemical processes to a larger extent than would be predicted from the temperature change alone. Two pilot studies indicate higher expression of heat shock genes after heating cells by MW exposure compared with conventional heating methods: one study in rats exposed to 1.7 W/cm^2^ (Walters et al. 1998) and the other in human glioma MO54 cells at an SAR of > 20 W/kg (Tian et al. 2002). These observations are in accordance with studies on MW-assisted chemistry, where the kinetics of chemical reactions is faster after exposure to high-power MWs compared with those observed when the same magnitude of heating is obtained by conventional methods (Kappe 2004; Stuerga 2006). MW-assisted chemistry is now widely used for a variety of applications, notably in organic chemistry [reviewed by Collins and Leadbeater (2007), Kappe (2004), and Lidström et al. (2001)]; it has also been used for chemistry of nucleic acid in ionic buffers (Orrling et al. 2004). No mechanism has been identified to date for an MW-specific effect, if any, in solutions. Yet, the specificity of MW heating is its in-core volumetric nature, and most authors agree about a possible role for energy absorption at the precise place of the reacting molecules. When considering, for example, the local SAR value *in vivo* at the interface between free DNA and the surrounding solution, a very preferential MW energy absorption exists at that place because of the particularly high concentrations of bound water molecules and counterions at this interface (Vanderstraeten and Vander Vorst 2004). As a possible explanation for the phenomenon of MW-assisted chemistry, and based on a quantum mechanical model of an S_N_2 reaction, Kalhori et al. (2002) suggested that bound water molecules could confer vibrational modes in the low-GHz frequency range to solvated reaction complexes. In addition, a local superheating effect (temperature hot spots) has been proposed as a mechanism (Stuerga 2006). However, using a standard heat-conduction model, Laurence et al. (2003), estimated that space-averaged SAR of the order of hundreds of watts per kilogram would lead to a local temperature gradient of only femto degrees (°C) in DNA-sized structures where significant SAR hot spots yet exist, relative to the surrounding medium. Therefore, no precise mechanism has been identified to date. If a phenomenon of MW-assisted chemistry was established in *in vitro* or *in vivo* systems using a nonthermal regime, no precise response pattern, if any, could be predicted after MW exposure. This response would depend not only on the functional status of the exposed cell and, presumably, of the heat-sensitive nature of the biochemical processes in progress, but also on the value of the time-averaged SAR and, for the same SAR value, on the duty cycle, which reflects the part of total exposure time during which an effective energy supply takes place. Although threshold values of these last parameters remain to be determined, it is uncertain whether this hypothesis would apply to the particular exposure to RF fields from current cellular phones, because SAR values often do not exceed a few tenths of watts per kilogram for the GSM phones, for example (Wiart et al. 2000).

## Conclusions

Because the overall results from the currently available literature are inconclusive and, in particular, because most of the reported positive findings are flawed by methodologic imperfections or shortcomings, uncertainty still prevails about the possible influence on gene and protein expression from RF exposure at intensities relevant to usual human health. Yet, from theoretical as well as experimental arguments, it is uncertain whether any specific gene or protein response pattern could ever be established after exposure to RF fields from mobile phones. In any case, further studies using HTSTs in this field should meet criteria as much as possible, allowing for unequivocal interpretation of the results (Corvi et al. 2006; Mayo et al. 2006). Also, because of possible different responses according to cell type, as suggested by Remondini et al. (2006) and Nylund and Leszczynski (2006), studies should further compare different biosystems and in particular, steady-state systems with those where a constitutive and specific gene overexpression exists. Finally, following the example of Chauhan et al. (2007) and Qutob et al. (2006), different exposure intensities should be further compared to assess a possible SAR threshold value, if any, above which a gene and/or protein response might be observed after exposure to RF fields in the frequency range relevant to human exposure. The biological relevancy of any response must then be confirmed by related phenotypic changes.

## Figures and Tables

**Table 1:** Studies addressing gene and protein expression with use of HTSTs.

		Exposure			Test				Response		
Reference	Cell or tissue^a^	Signal^b^ (MHz)	SAR (W/kg)^c^	Duration	End point (*n*)^d^	Pl^e^	No.^f^	Conf^g^	Fold^h^	No.^i^	Type^j^
Harvey and French 1999	HMC-1 mast cells	CW 864	7.3	3 × 20′/day 7 days	GE (558)	Mb	2	No	× 1.25	3	c-kit, NDPK, DAD-1
Leszczynski et al. 2002	EA hy926 endothelial cells	GSM 900	2.0	1 hr	PP (1,200)		1	Yes*		391	Unspecified (^¬^/HSP27*)
Pacini et al. 2002	Skin fibroblasts	GSM 902	0.6	1 hr	GE (91)	Mb	1	No	× 1.5	14	Stress, cc regulators
Port et al. 2003	HL-60 leukemia cells	PMW 400	50 kV/m	6 min	GE (1,176)	Mb	2	No	× 2.0	0	
Leszczynski et al. 2004	EA hy926 cells	GSM 900	2.4	1 hr	PE (1,300)		10	Yes*		49	Various (cytoskeleton*)
		GSM 900	2.4	1 hr	GE (3,600)	Mb	3	No		?	Fas/TNF-α, apo
Nylund and Leszczynski 2004	EA.hy926 cells	GSM 900	2.4	1 hr	PE (1,300)		10	Yes*		38	Various (cytoskeleton*)
Lee et al. 2005	HL60 cells	PMW 2,450	10	2 hr 6 hr	GE	SAGE	1	No	× 4.0	221 759	met, apo, cc, tpt, RNAp-t, RNAp met, glia function
Belyaev et al. 2006	Rat cerebellum (*in vivo*)	GSM 915	0.4	2 hr	GE (8,800)	Oc	3	No	× 1.3	12	
Gurisik et al. 2006	SK-N-SH neuroblastoma cell	GSM 900	0.2	2 hr	GE (8,400)	Oc	1	Yes	× 1.3	0	
Huang et al. 2006	Jurkat T lymphocytes	CDMA 1,763	10	1 hr/day 7 days	GE (30,000)	Gl	5	No	?	68	apo, met
Qutob et al. 2006	U87MG glioblastoma cells	PMW 1,900	0.1, 1, 10	4 hr	GE (18,000)	Oc	5	Yes	× 2.0	0	
Whitehead et al. 2006	C3H 10T (1/2) mouse cells	FDMA 836	5.0	24 hr	GE (9,200)	Oc	3	No	× 1.3	0	
		CDMA 848	5.0	24 hr	GE (9,200)	Oc	3	No	× 1.3	0	
Zeng et al. 2006	MCF-7 breast cancer cells	GSM 1,800	2.0, 3.5	24 hr 5′ on/10′ off	GE (14,500)	Oc	2	Yes	× 1.2	0	
		GSM 1,800	3.5	1 to 24 hr (int/cont)	PE (1,100)		1	No	× 2.0	34	
Remondini et al. 2006	NB69 neuroblastoma cells	GSM 1,800	2.0	24 hr 5′on/5′off	GE (75,000)	Mb	3	No	× 1.2	0	
	T lymphocytes (quiescent)	GSM 1,800	1.4	44hr 10′on/20′off	GE (75,000)	Mb	3	No	× 1.2	0	
	EA.hy926 cells	GSM 900	1.8–2.5	1 hr	GE (75,000)	Mb	2	No	× 1.2	32	met, stress, sign, diff
		GSM 1,800	1.8–2.5	1 hr	GE (75,000)	Mb	4	No	× 1.2	0	
	HL-60 cells	GSM 1,800	1.0	24 hr 5′on/5′off	GE (75,000)	Mb	6	No	× 1.2	12	met, stress
		GSM 1,800	1.3	24 hr	GE (75,000)	Mb	6	No	× 1.2	0	
	U937 lymphoma monocytes	GSM 900	2.0	1 hr	GE (75,000)	Mb	5	No	× 1.2	34	met, signal, diff
	CHME5 microglial cells	GSM 900	2.0	1 hr	GE (75,000)	Mb	5	No		0	
Nylund and Leszczynski 2006	EA hy926 cells	GSM 900	2.8	1 hr	GE (1,167) PE	Mb	3 10	No	× 2.0	1 38	Unspecified
	EA hy926v1 cells	GSM 900	2.8	1 hr	GE (1,167) PE	Mb	3 10	No	× 2.0	13 45	Various Unspecified
Zhao et al. 2007	Rat neurons	GSM 1,800	2.0	24 hr 5′on /10′off	GE (> 1,200)	Oc	1	No	× 1.15	34	Various
Chauhan et al. 2007	U87MG glioblastoma cells	PMW 1,900	0.1, 1.0, 10	24 hr	GE (18,000)	Oc	5	Yes	× 1.35	0	
	MM6 monocytoid cells	PMW 1,900	1.0, 10	6 hr 5′on/10′off	GE (18,000)	Oc	5	Yes	× 1.35	0	

Abbreviations: ‘, minutes; apo, apoptosis-related genes; cc, cell-cycle; DAD-1, defender against cell death 1; diff, differentiation; GE, gene expression; Gl, glass cDNA microarray; int/cont, intermittent/continuous; Mb, membrane-based cDNA microarray; met, metabolism; NDPK, nucleoside diphosphate kinase; Oc, oligonucleotide chip-based microarray; PE, protein expression; Pl, platform; PP, protein phosphorylation; RNAp, RNA processing; RNAt, RNA translation; sign, signaling; stress, stress response; tpt, transport.

a All cells are of human origin except where indicated.

b Mobile phone signal and carrier frequency.

c Space- and time-averaged value of the SAR.

d Number of screened genes or proteins.

e Platforms used for gene expression study; all reported studies on protein expression used two-dimensional gel electrophoresis.

f Number of independent experiments (all are sham-controlled with the exception of the 6-hr exposure reported by Lee et al. (2005).

g Confirmation experiment.

h Fold change cutoff value.

i Number of statistically responding genes.

j Predominant functional classification of affected genes as reported by the authors

asterisks indicate confirmed proteins.
